# Zinc Sulfate Stress Enhances Flavonoid Content and Antioxidant Capacity from Finger Millet Sprouts for High-Quality Production

**DOI:** 10.3390/foods14152563

**Published:** 2025-07-22

**Authors:** Xin Tian, Jing Zhang, Zhangqin Ye, Weiming Fang, Xiangli Ding, Yongqi Yin

**Affiliations:** 1 College of Food Science and Engineering, Yangzhou University, Yangzhou 210095, China; dx120230241@stu.yzu.edu.cn (X.T.); mz120222090@stu.yzu.edu.cn (J.Z.); mz120242240@stu.yzu.edu.cn (Z.Y.); wmfang@yzu.edu.cn (W.F.); 2 College of Tourism and Culinary Science, Yangzhou University, Yangzhou 210095, China

**Keywords:** exogenous stress, antioxidant system, secondary metabolites, phenylpropanoid pathway, gene expression

## Abstract

The enhancement of flavonoid content and antioxidant capacity in plants remains a significant area of focus in the investigation of plant-derived functional foods. This study systematically investigated the impact of exogenous zinc sulfate (5 mM ZnSO_4_) stress on flavonoid content and antioxidant capacity in finger millet (*Eleusine coracana* L.) sprouts, along with its underlying molecular mechanisms. The results demonstrated that treatment with 5 mM ZnSO_4_ significantly increased the flavonoid content in sprouts, reaching a maximum value of 5.59 μg/sprout on the 6th day of germination. ZnSO_4_ stress significantly enhanced the activities of PAL, 4CL, and C4H, while also considerably upregulating the expression levels of flavonoid-biosynthesis-related genes. Physiological indicators revealed that ZnSO_4_ stress increased the contents of malondialdehyde, hydrogen peroxide, and superoxide anion in the sprouts, while inhibiting sprout growth. As a stress response, ZnSO_4_ stress enhances the antioxidant system by increasing antioxidant capacity (ABTS, DPPH, and FRAP), antioxidant enzyme activity (POD and SOD), and related gene expression (*POD*, *CAT*, and *APX*) in sprouts. This study provides experimental evidence for ZnSO_4_ stress to improve flavonoid accumulation and antioxidant capacity in finger millet sprouts and provides important theoretical and practical guidance for the development of high-quality functional foods.

## 1. Introduction

Finger millet (*Eleusine coracana* L.) is a significant drought-resistant crop, extensively cultivated and consumed across various regions [1,2]. The crop is rich in nutrients and contains diverse secondary metabolites such as flavonoids, anthocyanins, and total phenols [3,4]. Consequently, it has been recognized as a potential “super grain” by the National Research Council of the United States. In vitro bioactivity and gastrointestinal simulation studies have demonstrated that beverages derived from finger millet are highly beneficial for human health, with these health benefits attributed to the active compounds, particularly flavonoids [5]. Flavonoids, as secondary metabolites, exhibit various physiological activities [6], including the regulation of blood glucose [7], lipids, and blood pressure [8], as well as the inhibition and treatment of various cancers [9,10]. Flavonoids are not endogenously synthesized and must be acquired exogenously. Unfortunately, the flavonoid supplements commonly available on the market are primarily produced through chemical synthesis, raising concerns about their potential health risks. The natural flavonoid content in sources like finger millet is limited, ranging from 62.23 to 74.05 mg/100 g [11], which is insufficient to meet the health needs of the population. Therefore, enhancing the content of natural flavonoids in finger millet through biological pathways and developing functional health foods rich in natural flavonoids using finger millet as a raw material holds significant health and economic value.

Seed germination, an economically viable bioprocessing technique, has been extensively employed to enhance the bioavailability of nutrients and the content of bioactive compounds in plants [12]. The process initiates the seed’s endogenous enzyme system, thereby initiating a cascade of biochemical reactions. This leads to the degradation or transformation of macromolecules. Simultaneously, antinutritional factors are effectively reduced and significantly enhance the accumulation of diverse plant secondary metabolites [13,14]. For instance, among 17 types of plant sprouts after germination, the flavonoid content in these sprouts was significantly higher than that in the seeds [15]. Similarly, after the germination of barnyard millet grains, the biosynthesis of flavonoids was significantly enhanced through biotransformation while retaining basic nutrients, and its antioxidant capacity was also far superior to that of ungerminated grains [14]. Research indicates that the germination of finger millet seeds triggers the release of flavonoids with antioxidant properties, thereby inhibiting the proliferation of breast and colorectal cancer cells [16]. Furthermore, finger millet sprouts are utilized in various Indian culinary applications, including chaklis, porridge, and infant gruel [17]. Consequently, the exploration of novel functional foods and nutraceuticals utilizing finger millet sprouts to address specific health needs or consumer demands presents a promising research avenue. In addition, developing a simple method that promotes the nutritional content of chicken claw sprouts will help increase the market supply and consumer acceptance, thereby realizing the full potential of this nutrient-rich grain.

In recent years, abiotic stress has garnered significant attention as a crucial method for modulating the synthesis of plant secondary metabolites [18,19,20,21]. Despite the adverse effects of abiotic stress on plant growth and development, it can notably induce the biosynthesis of secondary metabolites in plants and enhance the antioxidant system. Tian et al. [22] found that NaCl stress inhibited the growth of soybean sprouts while promoting the biosynthesis of isoflavones in the sprouts and antioxidant enzyme activity (SOD, POD, and CAT). Rapornrerk [23] indicated that zinc sulfate stress inhibited the growth of broccoli sprouts, while significantly increasing the content and bioactivity of total GLs, total phenolics, and DPPH radical scavenging activity. Song et al. [24] found that ZnSO_4_ stress promoted the production of secondary metabolites in grapes, particularly the synthesis of total phenolic compounds. Interestingly, our preliminary research found that 5 mM ZnSO_4_ stress can significantly promote the synthesis of flavonoids in finger millet sprouts. However, to our knowledge, there are currently no studies on the effects of ZnSO_4_ stress on the flavonoid content and antioxidant capacity of finger millet sprouts.

In plants, the biosynthesis of flavonoids primarily relies on the phenylpropanoid pathway and the flavonoid biosynthesis pathway, which involve key enzymes such as phenylalanine ammonia-lyase (PAL), cinnamate 4-hydroxylase (C4H), 4-coumaroyl-CoA ligase (4CL), chalcone synthase (CHS), and chalcone isomerase (CHI), among others. These enzymes collectively constitute a highly regulated metabolic network, providing the foundation for flavonoid skeleton synthesis. During plant germination, key enzymes involved in flavonoid synthesis can be activated. Numerous studies have confirmed that exogenous stimuli can effectively enhance this pathway through various mechanisms, promoting flavonoid accumulation. For instance, UV-B treatment has been shown to specifically enhance the activity of key flavonoid metabolic enzymes (such as 4CL and C4H) during buckwheat germination while simultaneously upregulating the expression levels of the *PAL*, *C4H*, *4CL*, *CHS*, *CHI*, and *CHR* genes, thereby significantly increasing the flavonoid content in sprouts [18].The synergistic effect of exogenous hormones such as melatonin and ethephon primarily increases flavonoid accumulation in soybean sprouts by stimulating changes in the expression of related genes (*PAL*, *C4H*, *4CL*, *CHS*, *CHI*, *IFS*, *IFR*, and *CHR)* and promoting the synthesis of key enzymes (PAL, C4H, and 4CL) in the phenylpropanoid pathway and downstream flavonoid biosynthesis pathways [25]. These studies elucidate the differences in the molecular mechanisms of exogenous-stimuli-induced flavonoid biosynthesis in different species. They provide a crucial theoretical foundation and research framework for an in-depth exploration of the molecular mechanisms by which ZnSO_4_ stress enhances flavonoid accumulation in finger millet sprouts, focusing on enzymatic activity and gene expression levels.

This study represents the first investigation into the impacts of ZnSO_4_ stress on the antioxidant capacity and flavonoid accumulation in finger millet sprouts. The antioxidant capacity, activity, and gene expression of key enzymes involved in flavonoid biosynthesis and antioxidant pathways in finger millet sprouts under 5 mM ZnSO_4_ stress were systematically analyzed. The research results provide a new material source for the development of plant-based functional foods.

## 2. Materials and Methods

### 2.1. Cultivation of Finger Millet Sprouts

Finger millet seeds that were uniformly sized and free of damage were selected, and they were disinfected by soaking in a 1.0% (*v*/*v*) sodium hypochlorite solution for 15 min. Then, they were rinsed with distilled water until the pH was neutral and soaked in deionized water at 31 °C for 6 h. The seeds were distributed evenly in two germination boxes lined with gauze and germinated at 31 °C. During germination, different solutions were sprayed onto the two germination boxes every 12 h: (1) the control treatment was sprayed with distilled water, designated as CK; (2) the exogenous stress group was treated with 5 mM ZnSO_4_, designated as ZnSO_4_. The photoperiod was set to a 6.5/17.5 **h** light–dark cycle. At specific germination time points (2, 4, and 6 days), sprouts from both boxes were randomly sampled for index determination and data analysis.

### 2.2. Physiological Metabolism

Thirty sprouts were randomly selected at specific germination stages (2 d, 4 d, and 6 d) to measure length, fresh weight, and dry weight. A vernier caliper (Mitutoyo, Shanghai, China) was used to measure the length of the sprouts. An analytical balance (Mettle, Shanghai, China) was employed to determine their fresh and dry weights.

The soluble sugar and chloroplast pigment contents in the sprouts were determined according to the method of Zhang et al. [26]. Briefly, a 0.2 g sample of sprouts was homogenized in deionized water and incubated in a 50 °C water bath for 20 min. Following incubation, it was centrifuged, and the supernatant was collected. An aliquot of the supernatant was mixed with 3,5-dinitrosalicylic acid (DNS) and heated in a boiling water bath for 5 min. After the reaction, a UV–Vis spectrophotometer was used to measure the absorbance of the sample solution at 540 nm, with deionized water as a blank control for zero adjustment. Simultaneously, glucose standard solutions with concentration gradients of 0.1 mg/mL, 0.2 mg/mL, 0.3 mg/mL, 0.4 mg/mL, and 0.5 mg/mL were prepared and subjected to the same colorimetric reaction and absorbance measurement procedure. A standard curve was generated by plotting glucose concentration against absorbance. Finally, the reducing sugar content in the sprout sample was calculated based on the absorbance of the sample solution and the standard curve.

$$\mathrm{Soluble}\;\mathrm{sugar}\;\mathrm{content}(\mathrm{mg}/g)=\frac{C\times (V/a)}{m}\times n$$

where C represents the Soluble sugar content (mg) of the sample, which was measured according to the standard curve. V represents the extract volume (mL); a represents the volume of sample liquid absorbed during color development (mL); m represents the sample weight (g); and n represents the dilution factor.

The sprouts were triturated with acetone, and the supernatant was collected after centrifugation and adjusted to a final volume of 10 mL. Acetone served as the blank control. Absorbance values at 663 nm and 645 nm were measured and recorded as *A*_663_ and *A*_645_, respectively. The chlorophyll content in the sample was calculated using the following formula:

$$C_{a}(\mu g/g)=12.7\times A_{663}-2.69\times A_{645}$$

$$C_{b}(\mu g/g)=22.9\times A_{645}-4.86\times A_{663}$$

$$C_{total}(\mu g/g)=C_{a}+C_{b}$$

$$Chloroplast\;pigment\;content(\mu g/g)=\frac{C_{total}\times V\times n}{m}$$

where *V* represents the extract volume (mL); m represents the sample weight (g); and n represents the dilution factor.

The malondialdehyde (MDA), hydrogen peroxide (H_2_O_2_), and superoxide anion ( $O_{2}^{–•}$) content were measured with modifications based on the method proposed by Yin et al. [27]. For the MDA content, homogenates of leaf segments produced in 10% trichloroacetic acid (TCA) containing 0.65% 2-thiobarbituric acid (TBA) and heated at 100°C for 15 min were used to determine the amount of oxidative damage to lipids. The MDA concentration was estimated by subtracting the absorbance of the same TBA solution from the absorbance at 535 nm of the exact solution of the plant extract without TBA.

The supernatant was reacted with a titanium sulfate reagent. The precipitate was collected by centrifugation and dissolved in sulfuric acid, and the absorbance was measured at 415 nm, quantified using a standard curve for H_2_O_2_ content.

The sample was homogenized in phosphate-buffered saline and centrifuged, and the supernatant was collected. The supernatant was mixed with a hydroxylamine hydrochloride solution and incubated for 1 h. Subsequently, the absorbance was measured at 530 nm. A standard curve was generated using sodium nitrite as a standard to calculate the $O_{2}^{–•}$ content in the sample.

$$O_{2}^{-•}content(nmol/g)=C\times V/(m\times 62\times t)$$

where C represents the sodium nitrite concentration (μg/mL) determined from the standard curve, V denotes the total volume of the sample extract (mL), m signifies the fresh weight of the sample (g), 62 is the molar mass of $O_{2}^{-•}$ (μg/μmol), and t indicates the reaction time (h).

### 2.3. Flavonoid Contents

Flavonoid content was assayed with rutin as the standard [22]. Thirty fresh samples were obtained and macerated in methanol. The homogenate was centrifuged, and the supernatant was collected. The supernatant was then mixed with sodium nitrite, aluminum nitrate, and sodium hydroxide. After a 15 min incubation period, the absorbance was measured at 510 nm. A standard curve was generated using rutin, and the flavonoid content in the samples was calculated accordingly.

Flavonoid content(μg/sprout)=C×V×n/30

where C represents the rutin concentration (mg/mL) determined from the standard curve, *V* denotes the total volume (mL) of the sample extract, n signifies the dilution factor.

### 2.4. Antioxidant Capacity

The sample was completely homogenized in methanol, and the supernatant was collected after centrifugation. For the ABTS assay, an ABTS working solution was prepared by reacting ABTS with potassium persulfate for 12–16 h. Subsequently, the sample extract was mixed with the ABTS working solution, and the absorbance was immediately measured at 734 nm after reacting in the dark for 30 min [28]. For the 1,1-diphenyl-7-finitrophenylhydrazine DPPH assay, the sample extract was mixed with a 0.1 mM DPPH–methanol solution and reacted, and the absorbance was measured at 517 nm [28]. The FRAP was determined with reference to Du et al. [29].

### 2.5. Antioxidant Enzyme Activity

The activity assays for ascorbate peroxidase (APX), catalase (CAT), peroxidase (POD), and superoxide dismutase (SOD) were performed according to Yin et al. [30]. Fresh sprout samples were homogenized in pre-chilled extraction buffer on ice and centrifuged, and the supernatant was collected as the crude enzyme extract. SOD activity was defined as a 50% inhibition of nitro-blue tetrazolium photochemical reduction; APX, POD, and SOD activities were measured by absorbance changes at 290 nm, 470 nm, and 240 nm (0.1/min), respectively.

### 2.6. Flavone Synthase Activity

The samples were homogenized in ice-cold extraction buffer. The supernatant, representing the crude enzyme extract, was used for activity measurements. PAL, C4H, and 4CL activities were defined as changes per minute at 290 nm, 340 nm, and 333 nm, respectively, and were defined as U/g FW [31].

### 2.7. Expression Levels of Antioxidants or Flavonoid Synthesis Genes

Following flash-freezing of the sprout material in liquid nitrogen, total RNA was extracted according to the manufacturer’s protocol for the plant RNA Extraction Kit (RC411, Vazyme, Nanjing,China). Total RNA was reverse-transcribed into cDNA using the PrimeScript™ RT Master Mix Kit (R423, Vazyme, Nanjing, China). Subsequently, the cDNA template was amplified via PCR using gene-specific primers (Supplementary Table S1) and the Mix. The 2^−^^ΔΔCt^ comparative approach was utilized to ascertain the relative expression levels of genes.

### 2.8. Statistical Analysis

All experiments were performed in triplicate, and the results are presented as mean ± standard deviation (SD). Statistical analysis was performed using SPSS 21.0 software, including an unpaired t-test (CK and ZnSO_4_) and one-way analysis of variance (ANOVA) with Tukey’s test (different times). Statistical significance was set at *p* < 0.05 for all tests.

## 3. Results

### 3.1. Physiological Metabolism

As germination time increased, sprout length and fresh weight exhibited an increasing trend, whereas dry weight showed a decreasing trend. Specifically, compared to the control group, ZnSO_4_ stress significantly inhibited sprout growth at the same germination time and reduced the fresh weight of sprouts at 4 and 6 days of germination (Table 1, *p *< 0.05). However, exogenous ZnSO_4_ stress significantly increased the dry weight of sprouts under the same germination time (Table 1, *p* < 0.05).

The soluble sugar content of sprouts decreased with increasing germination time. ZnSO_4_ stress significantly increased the soluble sugar content in six-day-old sprouts, which was 2.16 times that of the control (Table 1). In addition, ZnSO_4_ stress significantly increased the chloroplast pigment content in four- and six-day-old sprouts, which were 27.21% and 24.77% higher than the control, respectively (Table 1).

**Table 1:** Effect of ZnSO_4_ stress on the physiologic index of sprouts.

Categories		Sprout’s Length (cm)	Fresh Weight (mg)	Dry Weight (mg)	Soluble Sugar Content (mg/g)	Chloroplast Pigment Content (μg/g)
CK	2 d	0.59 ± 0.09 ^c^*	69.75 ± 1.71 ^c^	21.75 ± 1.04 ^a^	9.80 ± 0.26 ^a^	83.21 ± 5.51 ^c^
	4 d	1.29 ± 0.13 ^b^*	120.53 ± 12.77 ^b^*	16.75 ± 1.10 ^b^	3.59 ± 0.05 ^b^	200.53 ± 2.54 ^a^
	6 d	1.37 ± 0.09 ^a^*	140.53 ± 8.50 ^a^*	13.50 ± 1.22 ^c^	0.99 ± 0.08 ^c^	177.43 ± 5.45 ^b^
ZnSO_4_	2 d	0.43 ± 0.09 ^b^	66.50 ± 5.25 ^b^	24.50 ± 0.58 ^a^*	9.18 ± 0.13 ^a^	78.26 ± 7.45 ^c^
	4 d	0.80 ± 0.13 ^ab^	97.52 ± 7.14 ^ab^	20.25 ± 2.10 ^b^*	3.96 ± 0.15 ^b^	219.57 ± 8.98 ^a^*
	6 d	1.01 ± 0.11 ^a^	103.01 ± 4.83 ^a^	16.75 ± 1.04 ^c^*	2.12 ± 0.04 ^c^*	195.19 ± 3.92 ^b^*

Note: Significant differences (*p* < 0.05) between different germination times under the same treatment are shown by different lowercase letters. * Indicates a significant difference (*p* < 0.05) between CK and ZnSO_4_ stress at the same time.

### 3.2. The Contents of MDA, H_2_O_2_, and $O_{2}^{–•}$

**Figure 1:**
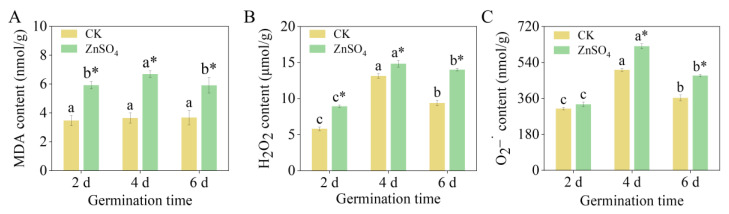
Effect of ZnSO_4_ stress on MDA content (**A**), H_2_O_2_ content (**B**), and $O_{2}^{–•}$ content (**C**) of sprouts. Significant differences (*p *< 0.05) between different germination times under the same treatment are shown by different lowercase letters. * Indicates a significant difference (*p *< 0.05) between CK and ZnSO_4_ stress at the same time.

The exogenous ZnSO_4_ treatment significantly increased the MDA and H_2_O_2_ contents, as well as the $O_{2}^{–•}$ content in the four-day-old and six-day-old sprouts (Figure 1, *p *< 0.05). Furthermore, the MDA, H_2_O_2_, and $O_{2}^{–•}$ contents in the four-day-old sprouts under ZnSO_4_ stress reached their maximum values of 6.69 nmol/g, 14.83 μmol/g, and 621.27 nmol/g, respectively, which were 83.01%, 12.94%, and 23.54% higher than those of the control sprouts at the same germination time. These results suggested that the finger millet sprouts were subjected to oxidative stress under ZnSO_4_ stress.

### 3.3. Flavonoid Content

**Figure 2:**
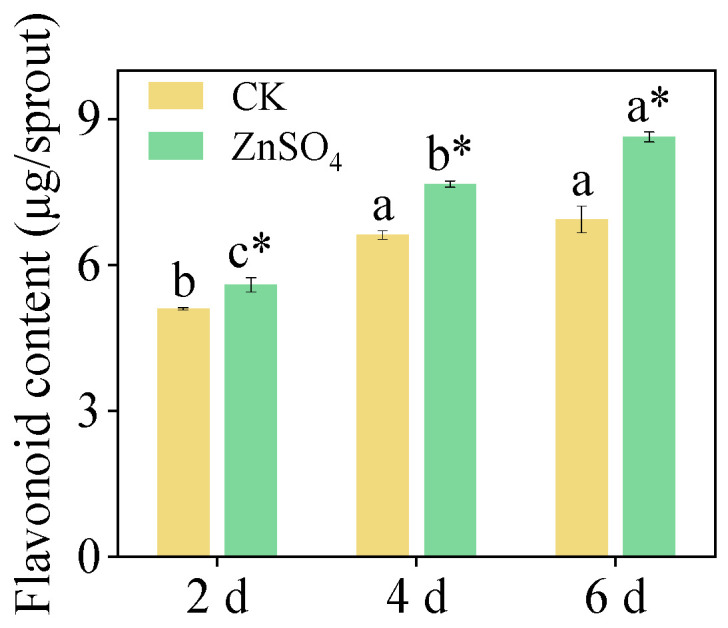
Effect of ZnSO_4_ stress on flavonoid content of sprouts. Significant differences (*p *< 0.05) between different germination times under the same treatment are shown by different lowercase letters. * Indicates a significant difference (*p *< 0.05) between CK and ZnSO_4_ stress at the same time.

Under identical germination conditions, the flavonoid content in the sprouts treated with ZnSO_4_ was significantly higher than that in the control group  (Figure 2, *p *< 0.05). The flavonoid content in the six-day-old sprouts treated with ZnSO_4_ reached a maximum of 5.59 μg/sprout, which was a 9.82% increase compared to the control group. These findings provided positive evidence that ZnSO_4_ stress increases the content of secondary metabolites in finger millet sprouts.

### 3.4. Antioxidant Capacity

**Figure 3:**
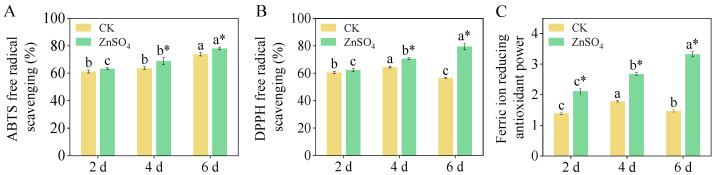
Effect of ZnSO_4_ stress on radical scavenging of ABTS (**A**), DPPH (**B**), and FRAP (**C**) of sprouts. Significant differences (*p *< 0.05) between different germination times under the same treatment are shown by different lowercase letters. * Indicates a significant difference (*p *< 0.05) between CK and ZnSO_4_ stress at the same time.

The ABTS free radical scavenging increased across all treatment groups with an extended germination time (Figure 3A). Compared to the control treatment, ZnSO_4_ stress significantly enhanced ABTS free radical scavenging in four- and six-day-old sprouts (*p* < 0.05), with increases of 8.30% and 5.72%, respectively. Furthermore, the ZnSO_4_ treatment significantly proved the DPPH free radical scavenging of four- and six-day-old sprouts (Figure 3B). Specifically, the DPPH free radical scavenging of six-day-old sprouts under ZnSO_4_ stress was 1.41 times that of the sprouts under the control treatment. The addition of exogenous ZnSO_4_ significantly increased the FRAP scavenging activity in sprouts, particularly in six-day-old sprouts, where it was 2.26 times higher than in the sprouts in the control group (Figure 3C).

### 3.5. Antioxidant Enzyme Activities

**Figure 4:**
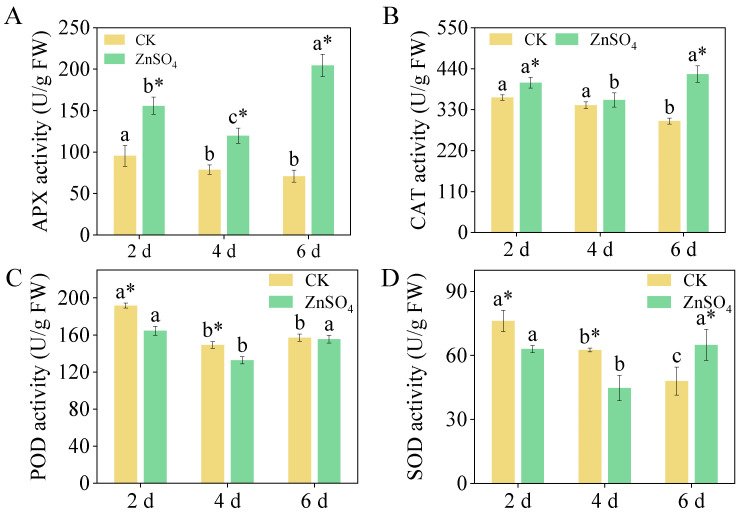
Effect of ZnSO_4_ stress on antioxidant enzyme ((**A**): APX, (**B**): CAT, (**C**): POD, and (**D**): SOD) activities in sprouts. Significant differences (*p *< 0.05) between different germination times under the same treatment are shown by different lowercase letters. * Indicates a significant difference (*p *< 0.05) between CK and ZnSO_4_ stress at the same time.

The ZnSO_4_ stress significantly enhanced the activity of APX, with APX activity reaching a maximum value of 155.78 U/g in six-day-old sprouts, which was 2.89 times higher than that in the control group  (Figure 4A). Under the control treatment, the CAT activity in six-day-old sprouts was significantly lower than that in two- and four-day-old sprouts, whereas ZnSO_4_ stress significantly increased CAT activity in two-day-old and six-day-old sprouts  (Figure 4B). Additionally, the ZnSO_4_ treatment significantly increased SOD activity in six-day-old sprouts, with activity levels 35.38% higher than the control group (Figure 4D).

### 3.6. Activities of PAL, C4H, and 4CL

**Figure 5:**
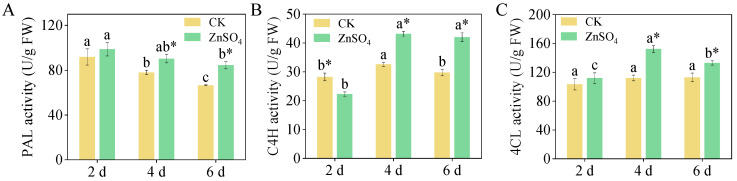
Effect of ZnSO_4_ stress on key enzyme activities ((**A**): PAL activity, (**B**): C4H activity, and (**C**): 4CL activity) in sprouts. Significant differences (*p *< 0.05) between different germination times under the same treatment are shown by different lowercase letters. * Indicates a significant difference (*p *< 0.05) between CK and ZnSO_4_ stress at the same time.

The data indicate that in the two-day-old sprouts, PAL activity was highest in both the control and ZnSO_4_-treated groups (Figure 5A). The addition of ZnSO_4_ significantly increased PAL, C4H, and 4CL activities in four- and six-day-old finger millet sprouts (*p* < 0.05). The addition of exogenous ZnSO_4_ did not significantly alter C4H activity in four- and six-day-old sprouts ( Figure 5B). After the control treatment, 4CL activity did not significantly differ in two-, four-, and six-day-old finger millet sprouts (Figure 5C). However, 4CL activity was highest in the four-day-old sprouts treated with ZnSO_4_, reaching 152.18 U/g, a 35.77% increase compared to the control group.

### 3.7. Gene Expression Levels

**Figure 6:**
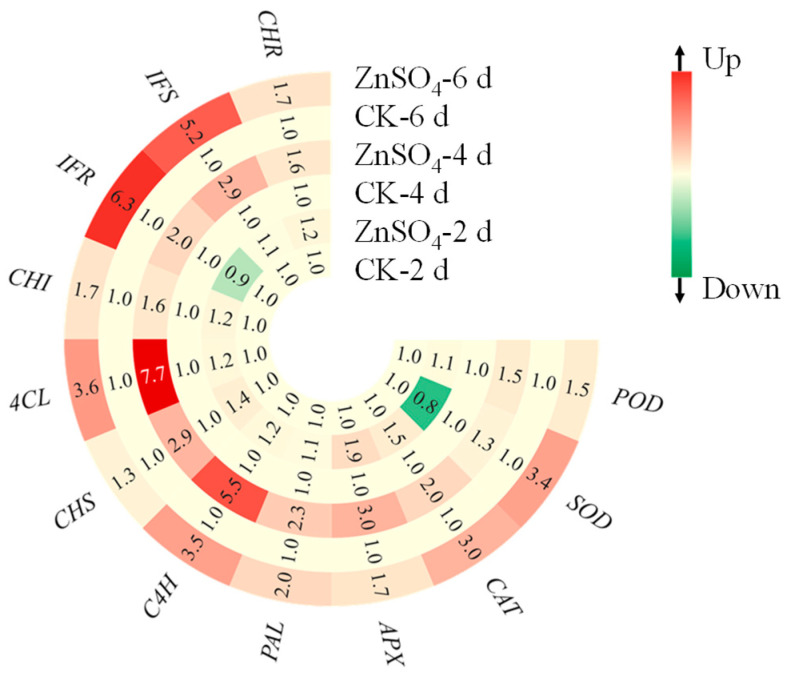
Effect of ZnSO_4_ stress on relative expression levels of genes in sprouts.

The results showed that ZnSO_4_ stress significantly increased the expression levels of *POD*, *CAT*, and *APX* in two-day-old sprouts and upregulated *POD*, *SOD*, *CAT*, and *APX* levels in four- and six-day-old sprouts  (Figure 6). Among these, the effect of ZnSO_4_ stress on the SOD expression level in 6-day-old seedlings was the most significant, increasing by 3.4 times. In addition, the ZnSO_4_ treatment markedly enhanced the expression level of *CHI*, *CHS*, and *CHR* in two-day-old sprouts, with respective increases of 1.2-, 1.4-, and 1.2-times. In the four- and six-day-old sprouts, the relative expression levels of flavonoid-biosynthesis-related genes (*PAL*, *C4H*, *CHS*, *4CL*, *CHI*, *IFR*, *IFS*, and *CHR*) were significantly elevated. Specifically, in the four-day-old sprouts, the ZnSO_4_ treatment exhibited the most pronounced effect on *4CL* expression levels. Conversely, in six-day-old sprouts, *IFR* expression levels were most strongly induced, with respective increases of 7.7-fold and 6.3-fold compared to the control. These findings provided compelling evidence that ZnSO_4_ treatment enhances flavonoid biosynthesis and the antioxidant system in finger millet sprouts.

## 4. Discussion

Finger millet, a drought-resistant staple crop originating from Africa, is widely cultivated in arid regions globally and is recognized as a superfood [1,2]. It is rich in bioactive compounds such as flavonoids [3,4], offering inherent advantages for the development of functional foods. Germination treatment can significantly enhance its nutritional efficacy by effectively degrading antinutritional factors and promoting the accumulation of secondary metabolites such as flavonoids [32,33,34], thereby substantially optimizing nutritional quality and processing suitability. Research indicates that the introduction of exogenous compounds can more effectively direct the enrichment of flavonoids compared to basic germination treatments. For instance, exogenous melatonin [35,36], methyl jasmonate [37], and calcium chloride [38] can significantly increase flavonoid biosynthesis in plants. Notably, our preliminary studies have shown that treatment with 5 mM ZnSO_4_ significantly enhances flavonoid biosynthesis in finger millet sprouts. However, the molecular mechanism is unclear. This study is the first to explore the effects of ZnSO_4_ treatment on the molecular mechanisms of flavonoid biosynthesis and antioxidant systems in finger millet sprouts to provide a theoretical basis for the development of functional foods with high flavonoid contents.

**Figure 7:**
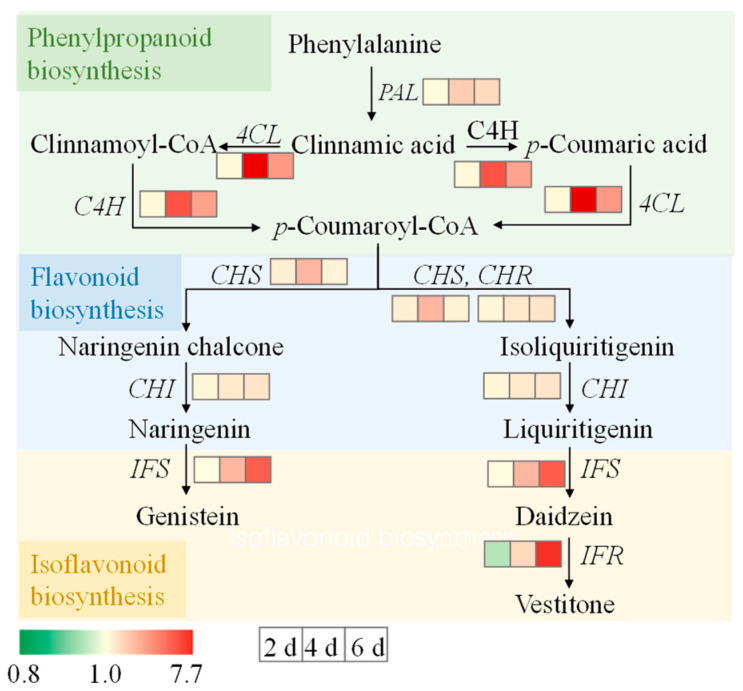
Flavonoid metabolic pathways in finger millet under ZnSO_4_ stress.

Flavonoids are important secondary metabolites in plants, and their content is often related to the activity of key enzymes in the phenylpropanoid metabolic pathway and the expression levels of related genes [39]. This pathway, central to flavonoid biosynthesis, initiates from phenylalanine and, through the catalysis of key enzymes such as PAL, C4H, and 4CL, ultimately produces flavonoids [40]. Numerous studies have demonstrated that exogenous compound treatments can modulate the accumulation of flavonoids in plants by regulating the activity of key enzymes and gene expression within the phenylpropanoid pathway [35,36,37,38]. Research on tomatoes revealed that methyl jasmonate application significantly enhanced the activity of PAL and CHS in the fruit, concurrently elevating the transcription levels of the genes encoding these enzymes, thereby markedly increasing the flavonoid content [41]. In water bamboo shoots, melatonin treatment activated the phenylpropanoid pathway, significantly boosting the activity of PAL, C4H, and 4CL and upregulating the expression of related genes, which promoted flavonoid accumulation [42]. Furthermore, in transgenic studies of licorice, the overexpression level of *PAL1* significantly enhanced flavonoid biosynthesis [43]. This study revealed that ZnSO_4_ stress significantly regulates flavonoid metabolism in finger millet sprouts (Figure 2 and Figure 7). ZnSO_4_ stress not only induced an increase in flavonoid content but also significantly elevated the activity of PAL, C4H, and 4CL (Figure 5 and Figure 7). Concurrently, ZnSO_4_ stress significantly upregulated the expression level of *PAL*, *C4H*, *CHS*, *4CL*, *CHI*, *IFR*, *IFS*, and *CHR* (Figure 6 and Figure 7). These genes encode key enzymes in the phenylpropanoid pathway and flavonoid synthesis branches, and their upregulated expression indicates that ZnSO_4_ stress ensures efficient flavonoid synthesis at the transcriptional level. Overall, ZnSO_4_ stress promotes a significant increase in flavonoid content in finger millet sprouts by activating key enzyme activity in the phenylpropanoid pathway and upregulating the expression level of related genes, thereby establishing a coordinated transcriptional and enzymatic regulation mechanism. However, the specific flavonoids and key genes in millet sprouts primarily influenced by ZnSO_4_ stress remain unidentified. Consequently, future investigations will utilize high-performance liquid chromatography–mass spectrometry for the identification and quantification of specific flavonoids during germination, alongside Western blotting to analyze the correlation between gene expression levels and the abundance of their corresponding enzyme proteins, thereby elucidating the direct impact of transcriptional regulation on enzyme activity.

The ZnSO_4_ treatment had a stress effect on the finger millet sprouts, as confirmed by the increased in MDA and H_2_O_2_ content (Figure 1A,B). The cell membrane, a critical site of environmental stress perception in plants, is highly susceptible to external factors. Numerous studies have demonstrated that adverse environmental conditions can disrupt membrane permeability, leading to metabolic dysfunction. For instance, salt stress significantly elevates MDA content in maize, causing oxidative damage [44], while heat stress increases MDA levels in grapes, inhibiting normal growth and development [45]. In response to environmental challenges, plants have evolved sophisticated antioxidant defense systems. Research has confirmed the efficacy of these systems; for example, under drought stress, wheat enhances drought resistance by increasing the activity of antioxidant enzymes such as SOD and POD, thereby maintaining cellular reactive oxygen species homeostasis [46]. Existing research has demonstrated that the overexpression of *IbDHAR1* enhances the activity of the related antioxidant enzyme, thereby improving the heat tolerance of potatoes [47]. This study further revealed that ZnSO_4_ stress significantly increased the activity of APX and CAT in the seedlings on the 2nd and 6th days (Figure 4). This may be attributed to APX, a key enzyme in the ascorbate–glutathione cycle, efficiently catalyzing the reaction of H_2_O_2_ with ascorbic acid to reduce H_2_O_2_ to water, while CAT directly decomposes intracellular H_2_O_2_ [48]. The synergistic action of APX and CAT effectively eliminates excess ROS within the cells. Concurrently, the relative expression levels of the corresponding genes for *APX* and *CAT* were significantly upregulated (Figure 6), indicating that sprouts actively respond to ZnSO_4_ stress at the transcriptional level. This response involves enhancing the expression of antioxidant enzyme genes, thereby increasing their activity and maintaining cellular redox balance.

In addition to the antioxidant enzyme system, secondary metabolites such as flavonoids play a critical role as defensive mechanisms for plants against environmental stresses. Flavonoids, with their unique polyphenol structure, exhibit remarkable free radical scavenging and oxidation-inhibiting activities. Studies have shown that under UV radiation, buckwheat accumulates a large amount of flavonoids, effectively reducing the damage of reactive oxygen species to cells, and the total flavonoid content is significantly positively correlated with antioxidant capacity [18]. Under salt stress, soybean plants rapidly synthesize flavonoid, enhancing their antioxidant capacity to resist external damage [49]. In this study, the flavonoid content in the sprouts significantly increased under ZnSO_4_ stress, and the antioxidant capacity also increased. This finding indicated that under ZnSO_4_ stress, sprouts not only enhance enzymatic defense by upregulating the expression of antioxidant enzyme genes but also enhance non-enzymatic antioxidant capacity by accumulating secondary metabolites such as flavonoids, synergistically responding to oxidative damage, and maintaining redox homeostasis within cells, further highlighting the complexity and efficiency of the plant’s antioxidant defense system. Furthermore, these results offered compelling evidence for the production of plant-based foods with health-promoting properties.

## 5. Conclusions

This study investigated the impact of ZnSO_4_ stress on the biosynthesis of flavonoids and the antioxidant system in finger millet sprouts. The results demonstrated that ZnSO_4_ stress primarily promotes flavonoid biosynthesis by enhancing the activity of key enzymes and the expression of related genes involved in the flavonoid biosynthetic pathway. Furthermore, the response measures of sprout stress effects caused by ZnSO_4_ stress were the enhancement of antioxidant enzyme activity, the increase in related gene expression levels, and the improvement of free radical scavenging ability. ZnSO_4_ stress represents a promising approach to enhancing the flavonoid content and antioxidant capacity of finger millet sprouts. This research provided novel insights for improving the functional components of plant sprouts and producing plant-based foods with health-promoting properties.

## Data Availability

The original contributions presented in this study are included in the article/Supplementary Materials. Further inquiries can be directed to the corresponding authors.

## Supplementary Materials

The following supporting information can be downloaded at: https://www.mdpi.com/article/10.3390/foods14152563/s1, Table S1: Sequence-specific primers used in the present study. Table S2: Exact values of all indicators in the manuscript.
