# Supplementary material for: Zinc Sulfate Stress Enhances Flavonoid Content and Antioxidant Capacity from Finger Millet Sprouts for High-Quality Production
Source: Foods. 2025 Jul 22;14(15):2563. doi: 10.3390/foods14152563 (PMC12346305; doi:10.3390/foods14152563)
Supplement: Supplementary file 1 [file foods-14-02563-s001.zip › foods-3732813-supplementary.pdf]

Supplementary Table S1. Sequence-specific primers used in the present study

Gene	Forward Primer (5' - 3')	Reverse Primer (5' - 3')
Actin	CTCACGCTCAAGECCCCAATC	GGCAACACGAAGCTCATTGEC
EcAPX	CACCTGTTCCTCGACTTTGC	T*EC*CGTTGCAGCAGTTGAGG
EcCAT	ACCCGCCTT*EC*C*EC*CTTTTT	CA*EC*GCCGAAAAGCATCCAT
EcPOD	CCAGGTGCTC*EC*CTCCGACGACC	GAGGTTGGTCATGGCGGCGAC
EcSOD	CTCC*EC*CGGCGACCTC*EC*CCAGC	CTGAGGCTGTCCTCCCTCCCTG
EcPAL	CGTGCCGCTCTCC*EC*CATTGC	CCTCTGCTGCGTTCACCTTGG
EcC4H	GACTTCCGCTTCCTGCCGTTC	CACGAGCTTGCCGACGATGAG
Ec4CL	GACGACAAGGCGACCAAGGC	CTCCACGCTGCTGATGTTCTCG
EcCHI	GCCGCCGTGGAGAAGTTCAAG	ACCGACGAGTCCTTGGAGAACG
EcCHR	AGTCTCAAGATCGCATGCTGGTG	AACTGTGGTGAGGTGTGCTGTG
EcCHS	ATGCTGTTCTCCGTCCCGAATTTC	CT*EC*TCTTCCTGGCGAGCACTTC
NEcIFS	AAGCAAGCGGATGTGGTGTTCTC	GCTCCAGTCACAGCCATATTCAG
EcIFR	CCTGGCTCGCCGTCAACAAG	GGATGCTGCTGGCTCTGCTG

Supplementary Table S2. Exact values of all indicators in the manuscript

**Categories**	CK			$ZnSO_4$		
	2 d	4 d	6 d	2 d	4 d	6 d
MDA content (nmol/g)	3.48 ± 0.35^{a}	3.65 ± 0.35^{a}	3.68 ± 0.50^{a}	5.92 ± 0.25^{b*}	6.69 ± 0.23^{a*}	5.92 ± 0.53^{b*}
H_2O_2 content (μmol/g)	5.81 ± 0.25^{c}	13.13 ± 0.33^{a}	9.39 ± 0.38^{b}	8.94 ± 0.19^{c*}	14.83 ± 0.46^{a*}	14.01 ± 0.19^{b*}
$O_2^{\bullet-}$ content (nmol/g)	309.27 ± 6.10^{c}	502.91 ± 7.62^{a}	362.48 ± 14.45^{b}	330.81 ± 11.30^{c}	621.27 ± 12.29^{a*}	474.67 ± 5.68^{b*}
Flavonoid content (μg/sprout)	5.10 ± 0.15^{b}	6.62 ± 0.09^{a}	6.94 ± 0.28^{a}	5.59 ± 0.15^{c*}	7.66 ± 0.07^{b*}	8.63 ± 0.10^{a*}
ABTS (%)	61.25 ± 1.13^{b}	63.64 ± 1.09^{b}	73.87 ± 1.21^{a}	63.30 ± 0.68^{c}	68.76 ± 2.47^{b*}	78.09 ± 1.04^{a*}
DPPH (%)	60.56 ± 0.89^{b}	64.49 ± 0.70^{a}	56.44 ± 0.23^{c}	62.28 ± 1.14^{c}	70.60 ± 0.76^{b*}	79.55 ± 2.34^{a*}
FRAP (%)	1.39 ± 0.03^{c}	1.78 ± 0.02^{a}	1.47 ± 0.04^{b}	2.11 ± 0.09^{c*}	2.67 ± 0.05^{b*}	3.32 ± 0.08^{a*}
APX activity (U/g)	95.46 ± 12.57^{a}	78.78 ± 5.63^{b}	70.82 ± 7.20^{b}	155.78 ± 10.44^{b*}	119.77 ± 9.20^{c*}	204.44 ± 13.46^{a*}
CAT activity (U/g)	362.49 ± 7.37^{a}	342.25 ± 9.18^{a}	299.39 ± 8.19^{b}	402.52 ± 14.47^{a*}	355.89 ± 19.13^{b}	425.72 ± 22.35^{a*}
POD activity (U/g)	191.74 ± 2.72^{a*}	149.20 ± 3.66^{b*}	157.08 ± 3.99^{b}	164.47 ± 4.95^{a}	132.78 ± 3.95^{b}	155.47 ± 4.08^{a}
SOD activity (U/g)	76.27 ± 5.02^{a*}	62.56 ± 0.90^{b*}	48.00 ± 6.56^{c}	63.03 ± 1.64^{a}	44.77 ± 5.91^{b}	64.98 ± 7.27^{a*}
PAL activity (U/g)	91.80 ± 7.16^{a}	78.01 ± 1.93^{b}	66.51 ± 0.32^{c}	98.69 ± 5.96^{a}	90.28 ± 3.72^{ab*}	84.46 ± 3.10^{b*}
C4H activity (U/g)	28.24 ± 1.36^{b*}	32.62 ± 0.72^{a}	29.73 ± 1.07^{b}	22.29 ± 0.76^{b}	43.16 ± 0.87^{a*}	42.02 ± 1.60^{a*}
4CL activity (U/g)	103.35 ± 7.85^{a}	112.09 ± 4.03^{a}	112.09 ± 4.03^{a}	112.09 ± 7.23^{c}	152.18 ± 4.67^{a*}	132.93 ± 2.88^{b*}

Note: Under the same indicator, * indicates that there are significant differences ($p<0.05$) in the different treatments under the same germination time; different lowercase letters indicate that there are significant differences ($p<0.05$) in the different germination times under the same treatment. CK: control group; ZnSO4: 5 mM ZnSO4 stress; 2 d: two-day-old sprouts; 4 d: four-day-old sprouts; 6 d: six-day-old sprouts.
